# The educational gradient in cardiovascular risk factors: impact of shared family factors in 228,346 Norwegian siblings

**DOI:** 10.1186/s12889-017-4123-0

**Published:** 2017-03-30

**Authors:** Inger Ariansen, Laust Hvas Mortensen, Sidsel Graff-Iversen, Hein Stigum, Marte Karoline Råberg Kjøllesdal, Øyvind Næss

**Affiliations:** 1 Domain for Mental and Physical Health, Norwegian Institute of Public Health, PO box 4404, Oslo, N-0403 Norway; 2 Section of Social Medicine, University of Copenhagen, PO box 2099, Copenhagen, K 1014 Denmark; 3 Department of Community Medicine, The Arctic University of Norway, Tromsø, Norway; 4 Institute of Health and Society, University of Oslo, PO box 1130, Oslo, 0318 Norway; 5 Division of Epidemiology, Norwegian Institute of Public Health, PO box 4404, Oslo, N-0403 Norway

**Keywords:** Socioeconomic position, Education, Cardiovascular disease risk factors, Family study, Siblings

## Abstract

**Background:**

Various indicators of childhood socioeconomic position have been related to cardiovascular disease (CVD) risk in adulthood. We investigated the impact of shared family factors on the educational gradient in midlife CVD risk factors by assessing within sibling similarities in the gradient using a discordant sibling design.

**Methods:**

Norwegian health survey data (1980–2003) was linked to educational and generational data. Participants with a full sibling in the health surveys (228,346 individuals in 98,046 sibships) were included. Associations between attained educational level (7–9 years, 10–11 years, 12 years, 13–16 years, or >16 years) and CVD risk factor levels in the study population was compared with the corresponding associations within siblings.

**Results:**

Educational gradients in risk factors were attenuated when factors shared by siblings was taken into account: A one category lower educational level was associated with 0.7 (95% confidence interval 0.6 to 0.8) mm Hg higher systolic blood pressure (27% attenuation), 0.4 (0.4 to 0.5) mmHg higher diastolic blood pressure (30%), 1.0 (1.0 to 1.1) more beats per minute higher heart rate (21%), 0.07 (0.06 to 0.07) mmol/l higher serum total cholesterol (32%), 0.2 (0.2 to 0.2) higher smoking level (5 categories) (30%), 0.15 (0.13 to 0.17) kg/m^2^ higher BMI (43%), and 0.2 (0.2 to 0.2) cm lower height (52%). Attenuation increased with shorter age-difference between siblings.

**Conclusion:**

About one third of the educational gradients in modifiable CVD risk factors may be explained by factors that siblings share. This implies that childhood environment is important for the prevention of CVD.

**Electronic supplementary material:**

The online version of this article (doi:10.1186/s12889-017-4123-0) contains supplementary material, which is available to authorized users.

## Background

In high income countries, cardiovascular disease (CVD) risk factors are generally more prevalent among adults with lower socioeconomic position (SEP) [1, 2]. A substantial proportion of the inverse associations between SEP and CVD are driven by the higher prevalence of CVD risk factors by lower SEP [3, 4]. This socioeconomic gradient in CVD represent a potential for disease prevention, and it is of great policy interest to determine which periods during the life course are important for development of the cardiovascular disease risk factors; elevated blood pressure, disadvantageous serum lipid profile, obesity, tobacco use and physical inactivity [5, 6].

Childhood SEP, usually indicated by parental level of education or occupational class, is associated with adult CVD risk factors in several populations [6–10]. Proposed mechanisms for this association include both biological factors such as poor maternal nutrition and health, intrauterine growth retardation, poor growth in early childhood, obesity in adolescence and repeated childhood infections, and include also environmental factors such as health behaviour and psychosocial factors in the family and surroundings [5, 6, 10]. CVD risk factors might track from childhood to adulthood [11], but are also modifiable in adult age. Body height, in contrast, is a stable trait through adult life. In this context, sibling similarity in the educational gradient in height might serve as a strong indicator of shared family environment.

Indicators of childhood and adulthood SEP are likely to be highly correlated, and it is difficult to assess their independent effect on CVD risk [12, 13]. An alternative and potentially robust approach is to use a sibling comparison, where a model of the association between attained education and CVD risk factors within siblings can be compared with a cohort model not using the sibling approach [10, 12]. Although this approach is not directly comparable with a randomized controlled trial, it makes it possible to ask the question; what would the socioeconomic gradient in cardiovascular risk factors be if everyone experienced similar family environment in childhood. A sibling comparison will control for a number of family factors (genetic and environmental) as a way to quantify the impact of early life on associations between exposures and outcomes in adulthood [10, 12, 14–16]. Studies using this method have reported that part of the educational gradient in adult all-cause and CVD mortality was explained by factors shared by siblings [14–16].

Except for one study by Lawlor et al. analysing the within sibling-effect of education on adult body mass index (BMI) [17], no other studies have to our knowledge investigated the within sibling-effect of education on CVD risk factors. According to that study, the major part of the inverse association between educational attainment and adult BMI may be explained by family factors shared by siblings [17].

We investigated the educational gradient in CVD risk factors between siblings in a large sample from the Norwegian general population, being in their early 40s. We aimed to assess the impact of factors in early life shared by siblings on educational gradient in adulthood CVD risk factors (blood pressure, total cholesterol, heart rate, BMI, smoking).

## Methods

### Study population

**Fig. 1:**
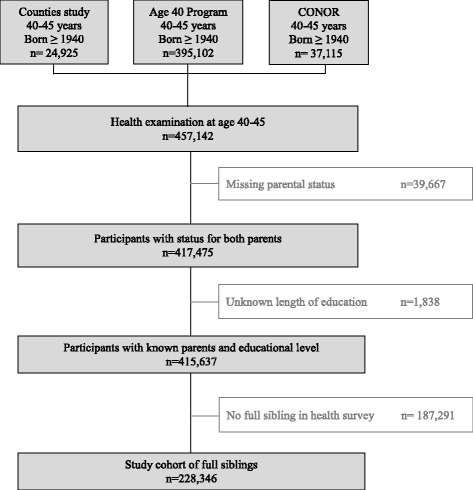
Participant flow diagram

Population based health survey participants from the Counties studies (1974-88) [18], the Age 40 Program (1985–1999) and CONOR (1994–2003) [19, 20], born after 1940, turning 40–45 years at the year of their health survey, and who had at least one full sibling among the health survey participants were selected for this sibling design. Participants with missing information for either/both of the parents, missing educational data and participants without any full siblings in the health surveys were excluded (Fig. 1).

The overall participation rate was 86% for the Counties study [21], 70% for the Age 40 Program [22] and 58% for CONOR [19].

### Data linkage

Health survey data were linked to national educational data, to the Norwegian Population Registry, and to the Norwegian Family Based Life Course (NFLC) study [23] using the unique national personal identification number.

### Generational data

The index person’s mother, father and siblings were identified from the NFLC study [23]. Parental identification has been proven to be reliable for index persons born from 1940 and onwards [23]. Full siblings were defined as persons registered with the same mother and father in the NFLC study. Twins were included.

### Education

Education was registered in the National Educational Database and reported in National Population and Housing Censuses every 10^th^ year from 1970–2001. A person’s highest attained educational level was classified as up to 9 years, [or 7 years in the 1960s], 10-11 years, 12 years (or vocational education with corresponding academic level), 13–16 years, and more than 16 years. Participants with no registered education were excluded.

### Cardiovascular risk factors

In all health surveys self-assessed questionnaires, clinical measures and non-fasting blood sampling were collected [18–20]. Smoking status and cigarette pack years were collapsed into a graded variable; 1) never smoker, 2) past smoker with < 20 pack years, 3) past smoker with ≥ 20 pack years, 4) current smoker with < 20 pack years, 5) current smoker with ≥ 20 pack years. Self-reported treatment with blood pressure lowering medications was recorded. Blood pressure was initially measured manually by sphygmomanometers (Ercameter, ERKA, Bad Tölz, Germany), and the second of two measurements defined systolic and diastolic blood pressure. Later, the average of the last two available automatic oscillometric measures (Dinamap, Criticon, Tampa, USA) [24] defined blood pressure. Heart rate, as a proxy for physical activity [25], was recorded during the automatic blood pressure measurements which were performed after 2 min of rest [24]. Height and weight were measured and BMI (kg/m^2^) was calculated. Non-fasting serum total cholesterol were initially measured by non-enzymatic, and later enzymatic method, and the non-enzymatic values were converted by a correction factor [26]. Systematic COronary Risk Evaluation (SCORE) risk prediction score of 10-year CVD mortality was calculated based on our variables; age, sex, systolic blood pressure, total cholesterol and current daily smoking. We applied the algorithm presented in Appendix A in the paper by Conroy et al. and chose the coefficients for CVD risk in a high risk population [27].

### Statistical analyses

Descriptive statistics are presented as mean ± standard deviation (SD) and as counts of persons (%). The Cuzick test for trend was used. Multilevel linear regressions of the exposure education was fitted in separate models for each of the outcomes; systolic blood pressure, diastolic blood pressure, heart rate, total cholesterol, BMI, height, smoking category and the SCORE risk score. The analyses were adjusted for sex, age at examination and examination year. The cohort estimate assesses the association between all individuals regardless of sibling similarities. In the within sibship estimate, each individual together with their sibling(s) made up a sibship cluster. The individual-specific factors that are constant in siblings (shared genes or familial environment) are omitted in the within sibships estimate. In addition, the within sibships term control for confounding from unobserved family-level factors. The dissimilarity of the association between education and the CVD risk factor for the cohort and the within sibships estimates was tested by using the Hausman specification test [28].

Evaluation of the dissimilarity between the cohort and within sibship estimates helps interpret the role of shared family factors. If the between- and within-associations are equal, it indicates that the unobserved family-specific factors in the within sibships analyses are not important. A weaker within sibships association than cohort association may indicate that these unmeasured family factors confound parts of the association. A stronger within sibships association may indicate that these unmeasured factors have obscured the association [28]. One example could be; among 4 siblings with different attained educational level, all overweight with a slightly lower BMI for the ones with higher educational level, there is a weaker within sibships educational gradient in BMI than among unrelated individuals in a cohort. If numerous sibships had such weaker gradients, the attenuation of the educational BMI gradient from the cohort analyses to the within sibships analyses would suggest that unmeasured family factors confound the association between educational level and BMI.

Variability expressed as SD in CVD risk factors between individuals in the cohort and within the sibships was assessed. The regression beta coefficient (β) represents the number of units more disadvantageous CVD risk factor per one lower educational level. The percentage change from the β_Cohort_ to the β_Within sibships_ was expressed as: % = ((β_Cohort_ – β_Within sibships_) / β_Cohort_)*100. In a sub-population of sibships with only two siblings, we stratified the population by age-difference between siblings.

Assumptions were examined in the cohort models by standard linear regression, for which diagnostic statistical tests are available. Potential deviations from linearity or non-constant variance were examined in plots of residuals against predicted values. Robustness was examined by plotting delta beta values against the participants’ reference numbers to look for points with high influence, and by plotting leverage-versus-squared-residual plots. We examined whether the cohort models gave negative predictions.

Sensitivity analyses were performed: We examined interaction between education and sex in the cohort models. We stratified the analyses by sex which meant that only same-sex siblings contributed. Also, the analyses were performed in a sub-population including only sibships with discordant educational level. The statistical package STATA version 13 was used.

## Results

### Study population

Of the 457,142 health survey participants born in 1940 and forth, and examined the year they turned 40–45 years, 91% had information on parental status from the NFLC study, while 39,667 individuals (9%) were excluded due to missing parental status (Fig. 1). The educational level was lower for those with missing parental status (21.7% basic education) than for those with registered parental status (16.3% basic education, *p* < 0.001). Of the participants with registered parental status 0.4% had unregistered length of education and were excluded from the analyses (Fig. 1). These excluded participants had similar mean percentage points SCORE risk score (0.59 ± 0.62 SD) as the included participants with registered length of education (0.62 ± 0.70), *p* = 0.496. Of the participants with registered parental status and registered length of education 45% did not have a full sibling in the health surveys and were excluded from the analyses (Fig. 1). For these excluded participants a lower proportion had basic education (15.1%) than for the included participants (17.4%), *p* < 0.001.

In all, 228,346 participants had a full sibling that had participated in one of the health surveys, and they defined the study population (Fig. 1).

### Descriptive analyses

The study population included 117,818 (52%) women and 110,528 (48%) men who were born from 1940–1963 and examined in 1980–2003 at median age 41 years. Only 1.2% of the study cohort had missing values for any of the CVD risk factors.

The study population of 228,346 participants made up 98,046 sibships that included up to 9 siblings. Of the sibships, 73% consisted of two siblings, 20% consisted of three siblings, and 6% consisted of four or more siblings. In all 164, 518 participants (72%) were parts of sibling groups with differing educational level between two or more siblings.

More disadvantageous CVD risk factor levels were found by lower levels of education in men and women (Table 1). Smaller variation and thus more similarity in CVD risk factors were found within sibships than between all individuals in the cohort (Table 2).

**Table 1:** Characteristics of the study cohort

	All educational levels	Years of attained education					
		7–9	10–11	12	13–16	>16	p trend
Men							
Participants, n (%)	110,528	18,348 (17)	34,889 (32)	31,351 (28)	18,593 (17)	7,347 (7)	
Age at examination, mean ± SD (years)	41.5 ± 1.1	41.5 ± 1.2	41.5 ± 1.1	41.4 ± 1.1	41.5 ± 1.1	41.6 ± 1.2	
Year of birth, mean	1951	1950	1951	1952	1952	1951	
Systolic blood pressure, mean ± SD (mmHg)	134 ± 13	135 ± 14	134 ± 14	133 ± 13	132 ± 13	132 ± 13	<0.001
Diastolic blood pressure, mean ± SD (mmHg)	80 ± 10	81 ± 10	80 ± 10	79 ± 10	79 ± 10	79 ± 9	<0.001
Heart rate, mean ± SD (beats/min)	72 ± 13	75 ± 13	73 ± 13	72 ± 12	70 ± 12	69 ± 12	<0.001
Total cholesterol, mean ± SD (mmol/l)	5.8 ± 1.1	6.0 ± 1.2	5.9 ± 1.1	5.8 ± 1.1	5.7 ± 1.0	5.6 ± 1.0	<0.001
BMI, mean ± SD (kg/m^2^)	25.7 ± 3.2	26.0 ± 3.5	25.8 ± 3.3	25.8 ± 3.1	25.3 ± 2.9	24.9 ± 2.8	<0.001
Height, mean ± SD (cm)	179 ± 6	178 ± 6	179 ± 6	179 ± 6	180 ± 6	181 ± 6	<0.001
Daily smokers, n (%)	44,063 (40)	9,998 (55)	15,637 (45)	12,412 (40)	4,877 (26)	1,139 (16)	<0.001
SCORE 10 year CVD mortality risk score, mean ± SD (percentage points)	1.1 ± 0.7	1.3 ± 0.8	1.2 ± 0.7	1.1 ± 0.6	0.9 ± 0.6	0.9 ± 0.5	<0.001
Antihypertensive treatment, n (%)	2,499 (2.3)	566 (3.1)	866 (2.5)	657 (2.1)	323 (1.7)	87 (1.1)	<0.001
Myocardial infarction, stroke and/or diabetes, n (%)	1,572 (1.4)	401 (2.2)	506 (1.5)	411 (1.3)	216 (1.2)	38 (0.5)	<0.001
Women							
Participants, n (%)	117,818 (100)	21,292 (18)	52,487 (45)	17,161 (15)	24,793 (21)	2,085 (2)	
Age at examination, mean ± SD (years)	41.5 ± 1.1	41.5 ± 1.1	41.5 ± 1.1	41.4 ± 1.1	41.5 ± 1.2	41.6 ± 1.2	
Year of birth, mean	1952	1950	1951	1952	1952	1952	
Systolic blood pressure, mean ± SD (mmHg)	124 ± 14	127 ± 15	125 ± 14	123 ± 14	122 ± 13	121 ± 13	<0.001
Diastolic blood pressure, mean ± SD (mmHg)	75 ± 10	77 ± 10	75 ± 10	74 ± 10	73 ± 10	73 ± 9	<0.001
Heart rate, mean ± SD (beats/min)	77 ± 12	80 ± 13	77 ± 12	76 ± 12	75 ± 12	74 ± 12	<0.001
Total cholesterol, mean ± SD (mmol/l)	5.5 ± 1.0	5.7 ± 1.1	5.5 ± 1.0	5.4 ± 1.0	5.3 ± 0.9	5.2 ± 0.9	<0.001
BMI, mean ± SD (kg/m^2^)	24.3 ± 3.9	24.7 ± 4.2	24.5 ± 3.9	24.4 ± 3.8	23.9 ± 3.4	23.3 ± 3.1	<0.001
Height, mean ± SD (cm)	166 ± 6	165 ± 6	166 ± 6	166 ± 6	167 ± 6	168 ± 6	<0.001
Daily smokers, n (%)	47,255 (40)	12,289 (58)	22,822 (44)	6,427 (37)	5,408 (22)	309 (15)	<0.001
SCORE 10 year CVD mortality risk score, mean ± SD (percentage points)	0.16 ± 0.11	0.20 ± 0.14	0.16 ± 0.10	0.15 ± 0.09	0.13 ± 0.08	0.12 ± 0.07	<0.001
Antihypertensive treatment, n (%)	2,168 (1.8)	563 (2.7)	1,028 (2.0)	287 (1.7)	275 (1.1)	15 (0.7)	<0.001
Myocardial infarction, stroke, and/or diabetes, n (%)	1,044 (0.9)	226 (1.1)	484 (0.9)	150 (0.9)	174 (0.7)	10 (0.5)	<0.001

N(%) is number of individuals (in proportion of all individuals). SD is standard deviation. CVD is cardiovascular disease. SCORE is the Systematic COronary Risk Evaluation risk prediction score of 10-year cardiovascular mortality

**Table 2:** Variation in educational level and cardiovascular risk factors within sibships and between individuals in the cohort

	Cohort	Cohort	Within sibships
	Mean level	Standard deviation	Standard deviation
Educational level (1–5 levels)	2.5	±0.9	±0.7
Systolic blood pressure (mmHg)	129	±13	±6
Diastolic blood pressure (mmHg)	77	±9	±5
Heart rate (beats/min)	75	±12	±5
Total cholesterol (mmol/l)	5.6	±0.9	±0.6
BMI (kg/m^2^)	25.0	±3.1	±1.8
Height (cm)	172	±8	±4
Daily smoking (daily smoking coded as 0–1)	0.4	±0.4	±0.2
SCORE (percentage points)	0.6	±0.7	±0.2

Educational levels 1–5: (1) up to 7–9 years; (2) 10–11 years; (3) 12 years (4) 13–16 years; (5) >16 years

SCORE is the Systematic COronary Risk Evaluation risk prediction score of 10-year cardiovascular mortality

### Cohort and within sibling analyses

The cohort and within sibships analyses differed significantly by Hausman specification test by *p* < 0.001 for all CVD risk factors in Table 3. In the within sibships analyses the educational gradients were attenuated; by one third for blood pressure, total cholesterol, smoking category and SCORE risk score, by two fifths for BMI, by one fifth for heart rate, and by one half for the opposite gradient in adult height (Table 3).

**Table 3:** Cardiovascular risk factor levels according to level of education in the cohort and within sibships

Education	Cohort	Within sibships	Difference		Cohort	Within sibships	Difference	
Years	β_Cohort_ (95% CI)	β_Within sibships_ (95% CI)	β_Δ_ (95% CI)	%	β_Cohort_ (95% CI)	β_Within sibships_ (95% CI)	β_Δ_ (95% CI)	%
ᅟ								
	Systolic blood pressure (mmHg)				Diastolic blood pressure (mmHg)			
7–9	3.6 (3.2, 3.9)	2.5 (2.0, 2.9)			2.1 (1.9, 2.3)	1.4 (1.1, 1.7)		
10–11	2.3 (2.0, 2.6)	1.6 (1.2, 2.0)			1.2 (1.0, 1.4)	0.8 (0.5, 1.0)		
12	1.6 (1.3, 1.9)	1.0 (0.6, 1.4)			0.7 (0.5, 0.9)	0.4 (0.1, 0.7)		
13–16	0.2 (-0.1, 0.5)	-0.0 (-0.4, 0.4)			0.1 (-0.1, 0.3)	-0.0 (-0.3, 0.2)		
>16	0	0			0	0		
Per lower level	1.0 (0.9, 1.0)	0.7 (0.6, 0.8)	0.3 (0.2, 0.3)	-27	0.6 (0.5, 0.6)	0.4 (0.4, 0.5)	0.2 (0.1, 0.2)	-30
	Total cholesterol, (mmol/l)				Heart rate (beats/min)			
7–9	0.38 (0.36, 0.41)	0.24 (0.22, 0.28)			5.4 (5.1, 5.6)	4.2 (3.8, 4.6)		
10–11	0.26 (0.24, 0.28)	0.19 (0.16, 0.22)			3.6 (3.3, 3.9)	2.9 (2.6, 3.3)		
12	0.20 (0.18, 0.22)	0.13 (0.10, 0.16)			2.6 (2.3, 2.9)	2.2 (1.8, 2.5)		
13–16	0.07 (0.05, 0.09)	0.04 (0.02, 0.07)			1.2 (0.9, 1.5)	0.9 (0.5, 1.3)		
>16	0	0			0	0		
Per lower level	0.10 (0.09, 0.10)	0.07 (0.06, 0.07)	0.03 (0.03, 0.04)	-32	1.3 (1.3, 1.4)	1.0 (1.0, 1.1)	0.3 (0.2, 0.3)	-21
	Body mass index, (kg/m^2^)				Height (cm)			
7–9	1.14 (1.06, 1.21)	0.61 (0.51, 0.72)			-2.1 (-2.2, -1.9)	-1.0 (-1.2, -0.9)		
10–11	0.88 (0.80, 0.95)	0.49 (0.39, 0.59)			-1.5 (-1.6, -1.4)	-0.8 (-1.0, -0.7)		
12	0.77 (0.69, 0.84)	0.45 (0.35, 0.55)			-1.3 (-1.4, -1.2)	-0.7 (-0.8, -0.6)		
13–16	0.31 (0.23, 0.38)	0.11 (0.02, 0.21)			-0.7 (-0.8, -0.6)	-0.4 (-0.5, -0.3)		
>16	0	0			0	0		
Per lower level	0.27 (0.25, 0.28)	0.15 (0.13, 0.17)	0.12 (0.10, 0.13)	-43	-0.4 (-0.5, -0.4)	-0.2 (-0.2, -0.2)	-0.2 (-0.2, -0.2)	52
	Smoking status and pack years (category)				SCORE (percentage points)			
7–9	1.30 (1.27, 1.34)	0.91 (0.86, 0.95)			0.31 (0.30, 0.32)	0.23 (0.22, 0.25)		
10–11	0.96 (0.93, 0.99)	0.71 (0.67, 0.75)			0.24 (0.23, 0.25)	0.20 (0.18, 0.21)		
12	0.81 (0.78, 0.84)	0.58 (0.53, 0.62)			0.20 (0.19, 0.21)	0.16 (0.15, 0.18)		
13–16	0.34 (0.31, 0.37)	0.25 (0.21, 0.29)			0.13 (0.12, 0.14)	0.12 (0.10, 0.13)		
>16	0	0			0	0		
Per lower level	0.31 (0.30, 0.31)	0.22 (0.21, 0.22)	0.09 (0.09, 0.10)	-30	0.06 (0.06, 0.07)	0.05 (0.04, 0.05)	0.02 (0.02, 0.02)	-30

β is the beta regression coefficient. 95% CI is 95% confidence interval. βΔ = β_Cohort_ – β_Within sibships_. % = ((β_Cohort_ – β_Within sibships_) / β_Cohort_)*100. Educational levels 1–5: (1) up to 7–9 years; (2) 10–11 years; (3) 12 years (4) 13–16 years; (5) >16 years. Smoking status and pack years categories 1–5: (1) never smoker, (2) past smoker and <20 pack-years, (3) past smoker and >20 pack-years, (4) current smoker and <20 pack-years, (5) current smoker and >20 pack-years). SCORE is the Systematic COronary Risk Evaluation risk prediction score of 10-year cardiovascular mortality. All analyses are adjusted for age at examination and examination year, both centred on median, and for sex. Analyses of blood pressure are adjusted for current antihypertensive treatment. The Hausman specification tests for difference between the cohort and within sibships effect estimates was significant *p* < 0.001 for all cardiovascular risk factors

In a subsample of sibships with only two siblings, analyses stratified for age-difference within a sibling pair indicated that a relatively larger proportion of the educational gradient in most of the CVD risk factors was explained by sibling similarities between siblings forin the sibling pairs born closer in time than for the sibling pairs with larger age-span (Table 4).

**Table 4:** Cardiovascular risk factor levels according to level of education in the cohort and within sibships stratified by age-difference in sibships of only two siblings

Sibling age-difference	Education	Cohort	Within sibships	Difference			Cohort	Within sibships	Difference		
Years	Level	β_Cohort_ (95% CI)	β_Within sibships_ (95% CI)	β_Δ_ (95% CI)	%	*p*	β_Cohort_ (95% CI)	β_Within sibships_ (95% CI)	β_Δ_ (95% CI)	%	*p*
	Per one lower level	Systolic blood pressure (mmHg)					Diastolic blood pressure (mmHg)				
0-1		0.79 (0.40, 1.18)	0.35 (-0.27, 0.97)	0.44 (-0.04, 0.92)	-56	<0.001	0.29 (-0.00, 0.56)	-0.07 (-0.51, 0.37)	0.35 (0.01, 0.69)	-126	<0.001
>1-6		1.00 (0.93, 1.07)	0.72 (0.60, 0.83)	0.28 (0.19, 0.37)	-28	<0.001	0.62 (0.56, 0.67)	0.44 (0.36, 0.52)	0.18 (0.11, 0.24)	-29	<0.001
>6		1.11 (0.95, 1.28)	0.95 (0.70, 1.21)	0.16 (-0.03, 0.36)	-14	<0.001	0.64 (0.53, 0.76)	0.54 (0.36. 0.72)	0.10 (-0.04, 0.24)	-16	<0.001
		Total cholesterol, (mmol/l)					Heart rate (beats/min)				
0-1		0.07 (0.04, 1.00)	0.01 (-0.03, 0.06)	0.06 (0.03, 0.09)	-81	<0.001	1.28 (0.92, 1.65)	0.76 (0.16, 1.37)	0.52 (0.04, 1.00)	-41	0.300
>1-6		0.10 (0.10, 0.11)	0.07 (0.06, 0.08)	0.03 (0.03, 0.04)	-31	<0.001	1.38 (1.31, 1.44)	1.12 (1.01, 1.22)	0.26 (0.18, 0.35)	-19	<0.001
>6		0.10 (0.09, 0.11)	0.07 (0.05, 0.09)	0.03 (0.02, 0.05)	-32	<0.001	1.32 (1.17, 1.47)	1.01 (0.77, 1.25)	0.32 (0.13, 0.50)	-24	0.004
		Body mass index, (kg/m^2^					Height (cm)				
0-1		0.19 (0.09, 0.30)	0.02 (-0.14, 0.17)	0.18 (0.06, 0.30)	-91	0.033	-0.49 (-0.66, -0.33)	-0.12 (-0.34, 0.10)	-0.37 (-0.53, -0.22)	75	<0.001
>1-6		0.29 (0.27, 0.31)	0.17 (0.14, 0.20)	0.12 (0.10, 0.14)	-42	<0.001	-0.49 (-0.52, -0.46)	-0.23 (-0.27, -0.18)	-0.26 (-0.29, -0.24)	54	<0.001
>6		0.30 (0.26, 0.35)	0.18 (0.11, 0.24)	0.13 (0.08, 0.18)	-42	<0.001	-0.46 (-0.53, -0.39)	-0.23 (-0.32, -0.14)	-0.23 (-0.29, -0.17)	50	<0.001
		Smoking status and pack years (category)					SCORE (percentage points)				
0-1		0.29 (0.25, 0.34)	0.18 (0.11, 0.24)	0.12 (0.06, 0.17)	-39	<0.001	0.061 (0.048, 0.074)	0.020 (-0.025, 0.042)	0.041 (0.024, 0.059)	-68	<0.001
>1-6		0.32 (0.32, 0.33)	0.23 (0.22, 0.24)	0.09 (0.08, 0.10)	-29	<0.001	0.065 (0.063, 0.068)	0.046 (0.043, 0.050)	0.019 (0.016, 0.022)	-29	<0.001
>6		0.30 (0.29, 0.32)	0.23 (0.21, 0.26)	0.07 (0.05, 0.09)	-23	<0.001	0.067 (0.061, 0.072)	0.054 (0.045, 0.063)	0.013 (0.006, 0.020)	-19	0.001

Number of individuals and number of sibships in sibships of only two siblings: 145,976 individuals in 72,988 sibships. Number of individuals and number of sibships stratified by difference in birth year: 4,208 individuals in 2,104 sibships for 0–1 years age-difference (of whom 2,540 individuals were born in the same year and month), 118,346 individuals in 59,173 sibships for >1–6 years age-difference, and 23,422 individuals in 11,711 sibships for >6 years age-difference. β is the beta regression coefficient. 95% CI is 95% confidence interval. βΔ = β_Cohort_ – β_Within sibships_. % = ((β_Cohort_ – β_Within sibships_) / β_Cohort_)*100. P is Hausman specification test p for difference between cohort and within sibships effect estimates. Educational levels 1–5: (1) up to 7–9 years; (2) 10–11 years; (3) 12 years (4) 13–16 years; (5) >16 years. Smoking status and pack years categories 1-5: (1) never smoker, (2) past smoker and <20 pack-years, (3) past smoker and >20 pack-years, (4) current smoker and <20 pack-years, (5) current smoker and >20 pack-years). SCORE is the Systematic COronary Risk Evaluation risk prediction score of 10-year cardiovascular mortality. All analyses are adjusted for age at examination and examination year, both centred on median, and for sex. Analyses of blood pressure are adjusted for current antihypertensive treatment

We found no important deviations from linearity, nor any important non-constant variance of residuals, nor any points with undue high influence. Negative predictions were found only for SCORE risk prediction score, and with no more than 7% negative predictions.

### Sensitivity analyses

Sex-stratified analyses included 73,023 sisters in 33,602 sibships and 65,400 brothers in 30,209 sibships, and showed a similar pattern as the main results. We did not formally test the sex differences in the estimates; however the educational gradients for blood pressure, total cholesterol and BMI in women were numerically stronger than in men (Additional file 1: Tables A and B). Analyses performed in the sub-population of only sibhips discordant for educational attainment showed similar pattern as in the main analyses (Additional file 1: Table C vs. Table 3).

## Discussion

In this study on average one third of the inverse educational gradients in blood pressure, serum total cholesterol, smoking, heart rate and BMI were explained by family factors shared by siblings. Furthermore, these educational gradients were explained to a greater extent with closer age-span between siblings.

The main strength of this study is the large sample size with clinical measurements, sex-stratified analyses, and the consistency of our finding across a wide range of CVD risk factors. The narrow age range of 40-45 years makes the risk factor levels comparable between siblings. The strict definition of siblings (with same mother and father) increases the plausibility of similar childhood environment and proportion of common genes. Also, the divorce rate was below 0.4% per year during 1940–1970 [29]. A potential weakness of the within sibships analysis is that it conditions on sibships discordant for education and CVD risk factors. This implies a selection of sibships that might differ in non-shared causes of the educational level attained and of the measured CVD risk factor level [30]. Non-shared environment include systematic elements such as birth order and birth year that might introduce non-shared confounding [10]. Our results were not altered when adjusting for birth order (results not shown), stratifying the analyses by sex, or restricting the analyses to sibships discordant for educational level (Additional file 1). Non-systematic non-shared environment that we did not take into account (season of birth, sibling-sibling interactions, differential parental treatment and peer groups) might also have contributed to non-shared confounding [10]. Our analyses were strengthened by including adult height, which is a composite measure of genetic disposition, health and nutrition during the growing years [31], is potentially modifiable during early life up to puberty where most siblings share environment, and is unlikely to be causally affected by attained education unlike the other CVD risk factors examined in this paper. Accordingly, shared family factors had the strongest impact on the educational gradient in height, of all the CVD risk factor gradients (Table 3). Our sibling design has thus the potential to capture unobserved shared confounding, as the educational gradient in height that is not explained by shared family factors might result from residual confounding from non-shared factors in early life, childhood and youth.

Our findings are consistent with the one previous study by Lawlor et al [17], comparing the associations between educational attainment and BMI within siblings of the same family and between individuals form different families in a smaller study comprising 5 467 individuals. Here the effect of education on adult BMI between individuals from different families was attenuated to the null in within sibling pair analyses.

The sibling similarities in the educational gradients in the modifiable midlife CVD risk factors, blood pressure, serum total cholesterol, smoking, heart rate and BMI, that we identify (Table 3), can be interpreted as a result of both the environment that siblings share; parents’ health behaviour, parents’ socioeconomic position, housing, neighbourhood and schooling, in addition to genes that full siblings share (approximately 50% [10]). These shared family similarities were weakened by a larger age-span between siblings (Table 4). This is in agreement with siblings with longer age–span sharing the same proportion of genes, but to a lesser extent their childhood environment than sibling pairs born closer in time [10], and suggests that family environment plays an important role in the socioeconomic gradient in CVD.

## Conclusions

Around one third of the association between lower educational attainment and less favourable risk factors seem to be explained by shared family factors. The contribution increases with shorter age-difference between siblings. This suggests that there is substantial scope for prevention of CVD that starts early and that childhood environment matters.

## Additional file

Additional file 1:
**Table A** Cardiovascular risk factor levels according to level of education in the cohort and within sibships in men. **Table B** Cardiovascular risk factor levels according to level of education in the cohort and within sibships in women. **Table C** Cardiovascular risk factor levels according to level of education in the cohort and within sibships in sibships discordant for educational level. (DOCX 46 kb)

## Abbreviations

BMI: Body mass index
CVD: Cardiovascular disease
NFLC study: The Norwegian family based life course study
SCORE: Systematic COronary risk evaluation
SD: Standard deviation
SEP: Socioeconomic position
β: Beta regression coefficient
