# Supplementary material for: The educational gradient in cardiovascular risk factors: impact of shared family factors in 228,346 Norwegian siblings
Source: BMC Public Health. 2017 Mar 30;17:281. doi: 10.1186/s12889-017-4123-0 (PMC5372258; doi:10.1186/s12889-017-4123-0)
Supplement: Additional file 1: — Table A Cardiovascular risk factor levels according to level of education in the cohort and within sibships in men. Table B Cardiovascular risk factor levels according to level of education in the cohort and within sibships in women. Table C Cardiovascular risk factor levels according to level of education in the cohort and within sibships in sibships discordant for educational level. (DOCX 46 kb) [file 12889_2017_4123_MOESM1_ESM.docx]

**Table A Cardiovascular risk factor levels according to level of education in the cohort and within sibships in men**

| **Education** | **Cohort** | **Within sibships** | | **Difference** | |  | **Cohort** | **Within sibships** | **Difference** | |  |
| --- | --- | --- | --- | --- | --- | --- | --- | --- | --- | --- | --- |
| Years | β_Cohort_ (95%CI) | β_Within sibships_ (95%CI) | β**_Δ_** (95%CI) | | % |  | β_Cohort_ (95%CI) | β_Within sibships_ (95%CI) | β**_Δ_** (95%CI) | % |  |
|  |  | | | |  |  |  | | |  |  |
|  | **Systolic blood pressure (mmHg)** | | | | |  | **Diastolic blood pressure (mmHg)** | | | |  |
| 7-9 | 2.7 (2.3, 3.2) | 1.9 (1.2, 2.6) |  | |  |  | 1.5 (1.1, 1.8) | 1.1 (0.6, 1.6) |  |  |  |
| 10-11 | 1.8 (1.3, 2.2) | 1.2 (0.6, 1.9) |  | |  |  | 0.9 (0.6, 1.3) | 0.7 (0.2, 1.2) |  |  |  |
| 12 | 1.2 (0.8, 1.7) | 0.9 (0.3, 1.5) |  | |  |  | 0.4 (0.1, 0.8) | 0.4 (-0.0, 0.9) |  |  |  |
| 13-16 | 0.3 (-0.2, 0.7) | 0.2 (-0.4, 0.8) |  | |  |  | 0.2 (-0.1, 0.6) | 0.2 (-0.3, 0.6) |  |  |  |
| >16 | 0 | 0 |  | |  |  | 0 | 0 |  |  |  |
| Per lower level | 0.7 (0.6, 0.8) | 0.5 (0.4, 0.6) | 0.2 (0.1, 0.3) | | -31 |  | 0.4 (0.3, 0.5) | 0.3 (0.2, 0.4) | 0.1 (0.0, 0.2) | -25 |  |
|  | **Total cholesterol, (mmol/l)** | | | | |  | **Heart rate (beats/min)** | | | |  |
| 7-9 | 0.33 (0.29, 0.37) | 0.21 (0.16, 0.26) |  | |  |  | 5.3 (4.8, 5.7) | 4.1 (3.8, 4.6) |  |  |  |
| 10-11 | 0.24 (0.21, 0.28) | 0.18 (0.13, 0.22) |  | |  |  | 3.7 (3.3, 4.1) | 2.9 (2.6, 3.3) |  |  |  |
| 12 | 0.20 (0.16, 0.23) | 0.14 (0.09, 0.18) |  | |  |  | 2.8 (2.4, 3.2) | 2.3 (1.8, 2.5) |  |  |  |
| 13-16 | 0.06 (0.02, 0.09) | 0.03 (-0.02, 0.08) |  | |  |  | 1.1 (0.6, 1.5) | 0.9 (0.5, 1.3) |  |  |  |
| >16 | 0 | 0 |  | |  |  | 0 | 0 |  |  |  |
| Per lower level | 0.08 (0.08, 0.09) | 0.06 (0.06, 0.07) | 0.03 (0.02, 0.04) | | -34 |  | 1.3 (1.2, 1.4) | 1.0 (0.9, 1.1) | 0.3 (0.2, 0.4) | -25 |  |
|  | **Body mass index , (kg/m^2^)** | | | | |  | **Height (cm)** | | | |  |
| 7-9 | 1.08 (0.97, 1.19) | 0.73 (0.51, 0.72) |  | |  |  | -2.3 (-2.5, -2.1) | -1.2 (-1.4, -0.9) |  |  |  |
| 10-11 | 0.85 (0.74, 0.95) | 0.55 (0.39, 0.59) |  | |  |  | -1.7 (-1.9, -1.5) | -0.9 (-1.1, -0.7) |  |  |  |
| 12 | 0.77 (0.66, 0.87) | 0.55 (0.35, 0.55) |  | |  |  | -1.4 (-1.6, -1.2) | -0.7 (-0.9, -0.5) |  |  |  |
| 13-16 | 0.30 (0.19, 0.41) | 0.16 (0.02, 0.21) |  | |  |  | -0.7 (-0.9, -0.5) | -0.4 (-0.6, -0.1) |  |  |  |
| >16 | 0 | 0 |  | |  |  | 0 | 0 |  |  |  |
| Per lower level | 0.25 (0.23, 0.27) | 0.17 (0.14, 0.20) | 0.09 (0.06, 0.11) | | -34 |  | -0.5 (-0.6, -0.5) | -0.3 (-0.3, -0.2) | -0.3 (-0.3, -0.2) | 49 |  |
|  | **Smoking status and pack years (category)** | | | | |  | **SCORE (percentage points)** | | | |  |
| 7-9 | 1.3 (1.2, 1.3) | 0.9 (0.8, 1.0) |  | |  |  | 0.43 (0.41, 0.45) | 0.29 (0.26, 0.33) |  |  |  |
| 10-11 | 1.0 (0.9, 1.0) | 0.7 (0.7, 0.8) |  | |  |  | 0.30 (0.28, 0.32) | 0.22 (0.19, 0.25) |  |  |  |
| 12 | 0.9 (0.8, 0.9) | 0.6 (0.6, 0.7) |  | |  |  | 0.24 (0.22, 0.26) | 0.17 (0.14, 0.20) |  |  |  |
| 13-16 | 0.4 (0.3, 0.4) | 0.3 (0.2, 0.3) |  | |  |  | 0.10 (0.07, 0.12) | 0.06 (0.03, 0.09) |  |  |  |
| >16 | 0 | 0 |  | |  |  | 0 | 0 |  |  |  |
| Per lower level | 0.3 (0.3, 0.3) | 0.2 (0.2, 0.2) | 0.08 (0.07, 0.09) | | -28 |  | 0.10 (0.10, 0.11) | 0.07 (0.07, 0.08) | 0.03 (0.03, 0.04) | -30 |  |
|  |  |  |  | |  |  |  |  |  |  |  |

The analyses include 65,400 men in 30,209 sibships. β is the beta regression coefficient. 95% CI is 95% confidence interval. βΔ = β_Cohort_ – β_Within sibships_. % = ((β_Cohort_ – β_Within sibships_ ) / β_Cohort_ )*100. Educational levels 1-5: (1) up to 7-9 years; (2) 10-11 years; (3) 12 years (4)13-16 years; (5) >16 years. Smoking status and pack years categories 1-5: (1) never smoker, (2) past smoker and <20 pack-years, (3) past smoker and >20 pack-years, (4) current smoker and <20 pack-years, (5) current smoker and >20 pack-years). SCORE is the Systematic COronary Risk Evaluation risk prediction score of 10-year cardiovascular mortality. All analyses are adjusted for age at examination and examination year, both centred on median, and for sex. Analyses of blood pressure are adjusted for current antihypertensive treatment. The Hausman specification tests for difference between the cohort and within sibships effect estimates was significant p<0.001 for all cardiovascular risk factors.

**Table B Cardiovascular risk factor levels according to level of education in the cohort and within sibships in women**

| **Education** | **Cohort** | **Within sibships** | | **Difference** | |  | **Cohort** | **Within sibships** | **Difference** | |  |
| --- | --- | --- | --- | --- | --- | --- | --- | --- | --- | --- | --- |
| Years | β_Cohort_ (95%CI) | β_Within sibships_ (95%CI) | β**_Δ_** (95%CI) | | % |  | β_Cohort_ (95%CI) | β_Within sibships_ (95%CI) | β**_Δ_** (95%CI) | % |  |
|  |  | | | |  |  |  | | |  |  |
|  | **Systolic blood pressure (mmHg)** | | | | |  | **Diastolic blood pressure (mmHg)** | | | |  |
| 7-9 | 4.7 (4.0, 5.6) | 3.0 (1.9, 4.1) |  | |  |  | 2.8 (2.3, 3.4) | 1.5 (0.7, 2.2) |  |  |  |
| 10-11 | 3.2 (2.4, 4.0) | 2.0 (1.0, 3.1) |  | |  |  | 1.7 (1.2, 2.3) | 0.8 (0.1, 1.5) |  |  |  |
| 12 | 2.2 (1.4, 3.0) | 1.3 (0.2, 2.4) |  | |  |  | 1.2 (0.7, 1.8) | 0.5 (-0.2, 1.2) |  |  |  |
| 13-16 | 0.7 (-0.1, 1.5) | 0.2 (-0.8, 1.2) |  | |  |  | 0.3 (-0.2, 0.9) | -0.1 (-0.8, 0.6) |  |  |  |
| >16 | 0 | 0 |  | |  |  | 0 | 0 |  |  |  |
| Per lower level | 1.3 (1.2, 1.4) | 0.9 (0.7, 1.0) | 0.4 (0.3, 0.5) | | -30 |  | 0.8 (0.7, 0.8) | 0.5 (0.4, 0.6) | 0.3 (0.2, 0.4) | -40 |  |
|  | **Total cholesterol, (mmol/l)** | | | | |  | **Heart rate (beats/min)** | | | |  |
| 7-9 | 0.46 (0.40, 0.51) | 0.29 (0.22, 0.37) |  | |  |  | 5.3 (4.6, 6.0) | 3.2 (2.1, 4.2) |  |  |  |
| 10-11 | 0.30 (0.25, 0.36) | 0.21 (0.14, 0.28) |  | |  |  | 3.4 (2.7, 4.1) | 1.9 (0.9, 2.8) |  |  |  |
| 12 | 0.23 (0.17, 0.28) | 0.13 (0.06, 0.21) |  | |  |  | 2.2 (1.5, 2.9) | 1.1 (0.2, 2.1) |  |  |  |
| 13-16 | 0.10 (0.05, 0.15) | 0.07 (0.00, 0.14) |  | |  |  | 1.2 (0.5, 1.9) | 0.5 (-0.5, 1.4) |  |  |  |
| >16 | 0 | 0 |  | |  |  | 0 | 0 |  |  |  |
| Per lower level | 0.11 (0.10, 0.12) | 0.07 (0.06, 0.08) | 0.04 (0.03, 0.05) | | -34 |  | 1.3 (1.2, 1.4) | 0.8 (0.7, 1.0) | 0.5 (0.3, 0.6) | -35 |  |
|  | **Body mass index , (kg/m^2^)** | | | | |  | **Height (cm)** | | | |  |
| 7-9 | 1.36 (1.14, 1.57) | 0.68 (0.39, 0.97) |  | |  |  | -2.0 (-2.3, -1.7) | -0.7 (-1.1, -0.4) |  |  |  |
| 10-11 | 1.09 (0.88, 1.30) | 0.62 (0.34, 0.90) |  | |  |  | -1.4 (-1.7, -1.1) | -0.6 (-0.9, -0.2) |  |  |  |
| 12 | 0.94 (0.72, 1.16) | 0.57 (0.29, 0.85) |  | |  |  | -1.2 (-1.5, -0.9) | -0.4 (-0.8, -0.1) |  |  |  |
| 13-16 | 0.49 (0.27, 0.70) | 0.28 (0.01, 0.55) |  | |  |  | -0.7 (-1.0, -0.4) | -0.2 (-0.5, 0.1) |  |  |  |
| >16 | 0 | 0 |  | |  |  | 0 | 0 |  |  |  |
| Per lower level | 0.29 (0.26, 0.32) | 0.14 (0.10, 0.18) | 0.15 (0.12, 0.18) | | -53 |  | -0.4 (-0.4, -0.4) | -0.2 (-0.2, -0.1) | -0.2 (-0.3, -0.2) | 57 |  |
|  | **Smoking status and pack years (category)** | | | | |  | **SCORE (percentage points)** | | | |  |
| 7-9 | 1.3 (1.2, 1.4) | 0.9 (0.7, 1.0) |  | |  |  | 0.08 (0.07, 0.08) | 0.05 (0.04, 0.06) |  |  |  |
| 10-11 | 0.9 (0.8, 1.0) | 0.6 (0.5, 0.7) |  | |  |  | 0.05 (0.04, 0.05) | 0.03 (0.02, 0.04) |  |  |  |
| 12 | 0.7 (0.7, 0.8) | 0.5 (0.4, 0.6) |  | |  |  | 0.04 (0.03, 0.04) | 0.02 (0.02, 0.03) |  |  |  |
| 13-16 | 0.3 (0.2, 0.4) | 0.2 (0.1, 0.3) |  | |  |  | 0.01 (0.01, 0.02) | 0.01 (0.00, 0.02) |  |  |  |
| >16 | 0 | 0 |  | |  |  | 0 | 0 |  |  |  |
| Per lower level | 0.3 (0.3, 0.3) | 0.2 (0.2, 0.2) | 0.1 (0.1, 0.1) | | -35 |  | 0.02 (0.02, 0.02) | 0.01 (0.01, 0.01) | 0.01 (0.01,0.01 ) | -36 |  |
|  |  |  |  | |  |  |  |  |  |  |  |

The analyses include 73,023 women in 33,602 sibships. β is the beta regression coefficient. 95% CI is 95% confidence interval. βΔ = β_Cohort_ – β_Within sibships_. % = ((β_Cohort_ – β_Within sibships_ ) / β_Cohort_ )*100. P is Hausman specification test p for difference between cohort and within sibships effect estimates. Educational levels 1-5: (1) up to 7-9 years; (2) 10-11 years; (3) 12 years (4)13-16 years; (5) >16 years. Smoking status and pack years (never smoker, past smoker and <20 pack-years, past smoker and >20 pack-years, current smoker and <20 pack-years, current smoker and >20 pack-years). Systematic COronary Risk Evaluation (SCORE) risk prediction score of 10-year cardiovascular mortality. All analyses are adjusted for age at examination and examination year, both centred on median, and for sex. Analyses of blood pressure are adjusted for current antihypertensive treatment.

| **Education** | **Cohort** | **Within sibships** | | **Difference** | |  | **Cohort** | **Within sibships** | **Difference** | | |  |
| --- | --- | --- | --- | --- | --- | --- | --- | --- | --- | --- | --- | --- |
| Years | β_Cohort_ (95%CI) | β_Within sibships_ (95%CI) | β**_Δ_** (95%CI) | | % |  | β_Cohort_ (95%CI) | β_Within sibships_ (95%CI) | β**_Δ_** (95%CI) | | % |  |
|  |  | | | |  |  |  | | | |  |  |
|  | **Systolic blood pressure (mmHg)** | | | | |  | **Diastolic blood pressure (mmHg)** | | | | |  |
| 7-9 | 3.4 (3.0, 3.7) | 2.5 (2.0, 2.9) |  | |  |  | 2.0 (1.8, 2.3) | 1.4 (1.1, 1.7) |  | |  |  |
| 10-11 | 2.4 (1.9, 2.6) | 1.6 (1.2, 2.0) |  | |  |  | 1.2 (1.0, 1.5) | 0.8 (0.5, 1.0) |  | |  |  |
| 12 | 1.6 (1.2, 1.9) | 1.0 (0.6, 1.4) |  | |  |  | 0.7 (0.5, 1.0) | 0.4 (0.1, 0.7) |  | |  |  |
| 13-16 | 0.3 (-0.0, 0.6) | -0.0 (-0.4, 0.4) |  | |  |  | 0.2 (-0.0, 0.4) | -0.1 (-0.3, 0.2) |  | |  |  |
| >16 | 0 | 0 |  | |  |  | 0 | 0 |  | |  |  |
| Per lower level | 0.9 (0.8, 1.0) | 0.7 (0.7, 0.8) | 0.2 (0.1, 0,2) | | -21 |  | 0.6 (0.5, 0.6) | 0.4 (0.4, 0.5) | 0.1 (0.1, 0.2) | | -26 |  |
|  | **Total cholesterol, (mmol/l)** | | | | |  | **Heart rate (beats/min)** | | | | |  |
| 7-9 | 0.36 (0.33, 0.38) | 0.25 (0.22, 0.28) |  | |  |  | 5.2 (4.8, 5.5) | 4.2 (3.8, 4.6) |  | |  |  |
| 10-11 | 0.25 (0.23, 0.28) | 0.19 (0.16, 0.22) |  | |  |  | 3.5 (3.2, 3.8) | 2.9 (2.5, 3.3) |  | |  |  |
| 12 | 0.19 (0.17, 0.22) | 0.13 (0.10, 0.16) |  | |  |  | 2.6 (2.3, 2.9) | 2.1 (1.8, 2.5) |  | |  |  |
| 13-16 | 0.07 (0.05, 0.10) | 0.04 (0.02, 0.07) |  | |  |  | 1.2 (0.9, 1.4) | 0.9 (0.5, 1.2) |  | |  |  |
| >16 | 0 | 0 |  | |  |  | 0 | 0 |  | |  |  |
| Per lower level | 0.09 (0.09, 0.09) | 0.07 (0.06, 0.07) | 0.02 (0.02, 0.03) | | -27 |  | 1.3 (1.2, 1.3) | 1.0 (1.0, 1.1) | 0.2 (0.2, 0.3) | | -18 |  |
|  | **Body mass index , (kg/m^2^)** | | | | |  | **Height (cm)** | | | | |  |
| 7-9 | 1.06 (0.97, 1.14) | 0.61 (0.51, 0.72) |  | |  |  | -1.8 (-2.0, -1.7) | -1.0 (-1.2, -0.9) |  | |  |  |
| 10-11 | 0.83 (0.76, 0.91) | 0.49 (0.40, 0.59) |  | |  |  | -1.4 (-1.5, -1.3) | -0.8 (-1.0, -0.7) |  | |  |  |
| 12 | 0.73 (0.65, 0.81) | 0.45 (0.35, 0.55) |  | |  |  | -1.2 (-1.3, -1.1) | -0.7 (-0.8, -0.6) |  | |  |  |
| 13-16 | 0.32 (0.24, 0.40) | 0.12 (0.02, 0.21) |  | |  |  | -0.7 (-0.8, -0.6) | -0.4 (-0.5, -0.3) |  | |  |  |
| >16 | 0 | 0 |  | |  |  | 0 | 0 |  | |  |  |
| Per lower level | 0.24 (0.23, 0.26) | 0.15 (0.13, 0.17) | 0.09 (0.08, 0.10) | | -37 |  | -0.4 (-0.4, -0.4) | -0.2 (-0.2, -0.2) | -0.02 (-0.02, -0.02) | | 44 |  |
|  | **Smoking status and pack years (category)** | | | | |  | **SCORE (percentage points)** | | | | |  |
| 7-9 | 1.25 (1.21, 1.28) | 0.90 (0.86, 0.95) |  | |  |  | 0.30 (0.29, 0.31) | 0.23 (0.22, 0.25) |  | |  |  |
| 10-11 | 0.94 (0.91, 0.97) | 0.71 (0.67, 0.75) |  | |  |  | 0.23 (0.22, 0.25) | 0.19 (0.18, 0.21) |  | |  |  |
| 12 | 0.79 (0.76, 0.83) | 0.58 (0.53, 0.62) |  | |  |  | 0.20 (0.19, 0.21) | 0.16 (0.15, 0.18) |  | |  |  |
| 13-16 | 0.35 (0.31, 0.38) | 0.24 (0.20, 0.28) |  | |  |  | 0.13 (0.12, 0.14) | 0.11 (0.10, 0.13) |  | |  |  |
| >16 | 0 | 0 |  | |  |  | 0 | 0 |  | |  |  |
| Per lower level | 0.29 (0.29, 0.30) | 0.21 (0.21, 0.22) | 0.08 (0.07, 0.08) | | -26 |  | 0.06 (0.06, 0.07) | 0.04 (0.04, 0.05) | 0.02 (0.02, 0.02) | | -28 |  |
|  |  |  |  | |  |  |  |  |  |  | |  |

**Table C Cardiovascular risk factor levels according to level of education in the cohort and within sibships in sibships discordant for educational level**

The analyses include 164,518 individuals in 68,169 sibships. β is the beta regression coefficient. 95% CI is 95% confidence interval. βΔ = β_Cohort_ – β_Within sibships_. % = ((β_Cohort_ – β_Within sibships_ ) / β_Cohort_ )*100. P is Hausman specification test p for difference between cohort and within sibships effect estimates. Educational levels 1-5: (1) up to 7-9 years; (2) 10-11 years; (3) 12 years (4)13-16 years; (5) >16 years. Smoking status and pack years (never smoker, past smoker and <20 pack-years, past smoker and >20 pack-years, current smoker and <20 pack-years, current smoker and >20 pack-years). Systematic COronary Risk Evaluation (SCORE) risk prediction score of 10-year cardiovascular mortality. All analyses are adjusted for age at examination and examination year, both centred on median, and for sex. Analyses of blood pressure are adjusted for current antihypertensive treatment.
